# Co-Targeting IGF-1R and Autophagy Enhances the Effects of Cell Growth Suppression and Apoptosis Induced by the IGF-1R Inhibitor NVP-AEW541 in Triple-Negative Breast Cancer Cells

**DOI:** 10.1371/journal.pone.0169229

**Published:** 2017-01-03

**Authors:** Weibin Wu, Jieyi Ma, Nan Shao, Yawei Shi, Ruiming Liu, Wen Li, Yin Lin, Shenming Wang

**Affiliations:** 1 Department of Breast Surgery, First Affiliated Hospital, Sun Yat-sen University, Guangzhou, China; 2 Department of Vascular Surgery, First Affiliated Hospital, Sun Yat-sen University, Guangzhou, China; 3 Laboratory of General Surgery, First Affiliated Hospital, Sun Yat-sen University, Guangzhou, China; University of South Alabama Mitchell Cancer Institute, UNITED STATES

## Abstract

**Background:**

Triple-negative breast cancer (TNBC) is the most intractable type of breast cancer, and there is a lack of effective targeted therapy. Insulin-like growth factor-1 receptor (IGF-1R) is reportedly a potential target for TNBC treatment. However, satisfying treatment outcomes in breast cancer patients have yet to be achieved with IGF-1R-targeted agents.

**Methods:**

To confirm whether inhibiting IGF-1R could induce autophagy, we detected autophagy-related proteins by western blotting and immunofluorescence staining of LC3-II. The IGF-1R inhibitor NVP-AEW541, autophagy inhibitor 3-methyladenine (3-MA) and Atg7 small interfering RNA (siRNA) were used to further investigate the effects of autophagy induced by IGF-1R inhibition in TNBC cells. The CCK8 assay, EdU assay, apoptosis and cell cycle analyses were applied to test cell function after treatment.

**Results:**

NVP-AEW541 markedly induced autophagy in TNBC cells by increasing the levels of the autophagy-related protein Beclin-1 and the LC3-II/LC-I ratio and reducing the selective autophagy substrate p62. Joint application of 3-MA or Atg7 siRNA enhanced the cell growth inhibition and apoptosis effects of NVP-AEW541 by arresting cells at G1/G0 phase and increasing Bax expression and decreasing that of Bcl-2.

**Conclusion:**

Targeting IGF-1R in TNBC induces cell-protective autophagy, thereby weakening the therapeutic effect of agents directed toward IGF-1R. Our findings reveal that combined use autophagy-disrupting agents can enhance the therapeutic efficacy of IGF-1R inhibitors in TNBC cells and may provide a valuable treatment strategy for IGF-1R inhibitor-based therapies for TNBC and other IGF-1 signaling-associated tumors.

## Introduction

Breast cancer is the second most prevalent cancer worldwide and according to an investigation by the World Health Organization, represents one of the leading causes of death in women cancer patients [1, 2].

Breast cancer can be divided into five major subtypes: luminal A, luminal B, Her-2-overexpressing, normal breast-like and basal-like subtypes. The majority of basal-like subtype tumors are triple-negative breast cancer (TNBC), which are highly malignant tumors. In this case, ‘triple negative’ indicates that no expression of estrogen-receptor (ER), progesterone-receptor (PR), and human epidermal growth factor receptor 2 (HER-2) is found in this type of breast cancer [3]. TNBC accounts for approximately 15% to 20% of all breast cancer cases and is usually associated with a relatively poor prognosis due to its aggressive behavior and the lack of effective targeting therapies compared with other subtypes [3]. Chemotherapy is currently the most common adjuvant treatment for TNBC. However, outcomes remain disappointing because of the high recurrence rate and the fact that only a minority of TNBC cases are actually chemosensitive [4]. Moreover, intrinsic or acquired resistance to chemotherapy also limits its efficacy and application [5, 6]. A number of genes have an important role in the establishment of drug tolerance, including BRCA1, TP53, PTEN, TGFBI, ING1, Bax, PinX1, APC, CDKN and BCRP/ABCG2 [7–10]. Autophagy has recently been found to be involved in the development of resistance to breast cancer therapies [11]. Although autophagy exhibits anti-tumor effects during tumorigenesis, it may contribute to the later development of cancer by promoting cancer cell survival and helping cancer cells to overcome stress during progression and metastasis as well as treatment [12]. Thus, using autophagy inhibitors alone or in combination with other cancer therapies may be a potential strategy for breast cancer treatment.

Insulin-like growth factor-1 (IGF-1) signaling is associated with various types of cancers, including pancreatic, lung and breast cancers [13–15]. Activation of IGF-1 receptor (IGF-1R) by IGF-1 binding results in cell proliferation, metastasis and drug resistance, and it is reported that IGF-1R promotes survival and proliferation of TNBC cell lines [16]. In fact, targeting IGF-1R inhibited migration and invasion of the TNCB cell line MDA-MB-231 [15]. Furthermore, in vivo experiments have shown that IGF-1R knockdown reduced the potential of MDA-MB-231 cells to establish brain metastases [17]. In view of these findings, inhibitors targeting IGF-1R may serve as antitumor agents, and several of them are currently undergoing clinical trials for various types of cancer [18]. Regardless, IGF-1R inhibitors have yet to be successfully translated into clinical medicine, possibly due to the complexity of IGF-1 signaling.

It has been revealed that down-regulation of IGF-1R stimulates the PI3K-Akt pathway, which is involved in cell autophagy. However, it remains unknown whether autophagy is responsible for the unsatisfactory outcomes of IGF-1R inhibitors in clinical trials. In the present study, we sought to investigate the effect of autophagy on TNBC cell lines in which IGF-1R has been inhibited and to clarify whether combining autophagy-disrupting agents can enhance the therapeutic efficacy of inhibitors that target IGF-1R in TNBC.

## Materials and Methods

### Cell lines and reagents

The human triple-negative breast cancer cell lines MDA-MB-231 and BT-549 were purchased from American Type Culture Collection (ATCC, Rockville, MD, USA) and cultured in Dulbecco’s modified Eagle medium (DMEM; Gibco, Karlsruhe, Germany) supplemented with 10% fetal bovine serum (FBS; Gibco, Karlsruhe, Germany) and 1% antibiotics (penicillin/streptomycin, Invitrogen, Carlsbad, CA, USA). The cells were maintained at 37°C in a humidified atmosphere containing 5% carbon dioxide.

NVP-AEW541 (IGF-1R inhibitor) was purchased from Selleck Chemicals (Selleck Chemicals, Houston, TX, USA). Rapamycin (mTOR inhibitor) was obtained from Cell Signaling Technology (CST; Beverly, MA, USA). 3-Methyladenine (3-MA; Autophagy Inhibitor) was obtained from Sigma-Aldrich Corporation (St Louis, MO, USA). All drugs were solubilized to produce a stock solution, and working dilutions were freshly prepared in culture medium before use.

Antibodies against the following were used for western blotting and immunofluorescence staining: total IGF-1R (#3027, Rabbit), p-IGF-1R^Tyr1316^ (#6113, Rabbit), total Akt (#4691, Rabbit), p-Akt^Ser473^ (#9271, Rabbit), Atg7 (#2631, Rabbit), Beclin-1 (#3495, Rabbit), SQSTM1/p62 (#5114, Rabbit), LC3B (#3868, Rabbit), GAPDH (#5174, Rabbit), Bax (#5023, Rabbit) and Bcl-2 (#2870, Rabbit); all were purchased from Cell Signaling Technology (Beverly, MA, USA). Appropriate secondary antibodies were obtained from ThermoFisher Scientific (Rockford, IL, USA).

### Western blotting analysis

Western blotting was conducted as previously described [19]. Proteins were extracted from lysates of MDA-MB-231 and BT-549 cells treated with the indicated concentration of drugs for 24 h. Protein concentrations were determined, and equal amounts of protein (30 μg) were loaded and separated by 10%-15% sodium dodecyl sulfate polyacrylamide gel electrophoresis (SDS-PAGE) under denaturing conditions. The proteins were then transferred onto polyvinylidene fluoride (PVDF) membranes (Millipore, Bedford, MA, USA). After blocking with 5% non-fat milk in Tris-buffered saline (TBS)/Tween 20, the membranes were incubated with primary antibodies overnight at 4°C. The membranes were washed and incubated with secondary horseradish peroxidase (HRP)-conjugated antibodies for 1 h at room temperature. Immunoreaction was visualized by enhanced chemiluminescence (ECL, Millipore, Bedford, MA, USA); images were obtained using a GE ImageQuant Las 4000 mini (GE, Fairfield, CT, USA).

### Immunofluorescence staining for LC3-II

MDA-MB-231 and BT-549 cells were grown overnight on coverslips in 24-well plates and treated with dimethyl sulfoxide (DMSO), NVP-AEW541 (1 μmol/L) or rapamycin (10 nmol/L) for 24 h. The cells were fixed with ice-cold paraformaldehyde for 15 min followed by membrane permeabilization using 0.1% Triton X-100 in phosphate-buffered saline (PBS) for 5 min. After blocking in 1% bovine serum albumin (BSA) for 1 h, the cells were incubated overnight with an anti-LC3 antibody at 4°C. The cells were then washed and incubated with Alexa Fluor 488 (Invitrogen, Carlsbad, CA, USA) for 1 h at room temperature, protected from light. Images were obtained by fluorescence microscopy and analyzed using Image-Pro Plus 6.0 software.

### Transfection of Atg7 small interfering RNA (siRNA)

Scrambled siRNAs and specific validated siRNAs for Atg7 were purchased from Applied Biosystems (Foster City, CA). Cells were seeded in the wells of culture dishes. After 24 h, the cells were transfected with 50 nmol/L scrambled siRNAs or 50 nmol/L Atg7 siRNA using the Lipofectamine RNAiMAX transfection reagent (Invitrogen, Carlsbad, CA, USA) according to the manufacturer’s protocol.

### Cell proliferation assays

Cell proliferation was measured using cell counting kit-8 (CCK-8; Dojindo, Kumamoto, Japan) and EdU (RiboBio, Guangzhou, China) assays. Cells were seeded in 96-well plates (5×10^3^ cells/well) and cultured overnight at 37°C, followed by treatment with DMSO, NVP-AEW541 (1 μmol/L) and/or 3-MA (5 mmol/L) for 24 h or transfection of Atg7 siRNA. For the CCK-8 assay, 10 μl of CCK-8 solution was added to each well, and the cells were incubated at 37°C for 2 h. The absorbance was measured at 450 nm, as previously described [20]. For the EdU assay, cells were exposed to EdU (50 μmol/L) for 2 h at 37°C and then fixed in 4% formaldehyde for 30 min. The cells were subsequently treated with Apollo cocktail for 30 min, followed by nuclear staining with Hoechst33342 for 30 min. Images were visualized by inverted fluorescence microscopy (Carl Zeiss Axio Observer Z1, Jena, Germany) and analyzed using Image-Pro Plus 6.0 software.

### Apoptosis and cell cycle analysis

Apoptosis and the cell cycle distribution were measured by cytometry analysis. For the apoptosis assay, cells were seeded in 6-well plates and incubated overnight at 37°C. After drug treatment or Atg7 transfection, the cells were harvested and stained using an Annexin V-FITC apoptosis kit (Dojindo, Kumamoto, Japan) according to the manufacturer’s instructions and then analyzed by flow cytometry (BD Bioscience, USA). For cell cycle analysis, cells subjected to the indicated treatments were harvested and fixed overnight at -20°C with 70% ethanol. The cells were then stained with propidium iodide (50 μg/mL) with Rnase I (100 μg/mL) and 0.1% Triton X-100 in PBS at 37°C for 30 min. Cell cycle analysis was performed by flow cytometry (BD Bioscience, USA).

### Statistics

Data are expressed as the means ± SD. Differences between groups were assessed by Student’s t-test and one-way ANOVA followed by Dunnett’s multiple comparison test. GraphPad Prism version 5.0 (GraphPad, San Diego, CA, USA) and SPSS version 17.0 (SPSS Inc., Chicago, IL, USA) were used for all of the statistical calculations. Results with p values <0.05 were considered statistically significant.

## Results

### Targeting IGF-1R inhibited TNBC cell proliferation by attenuating the Akt pathway

**Fig 1:**
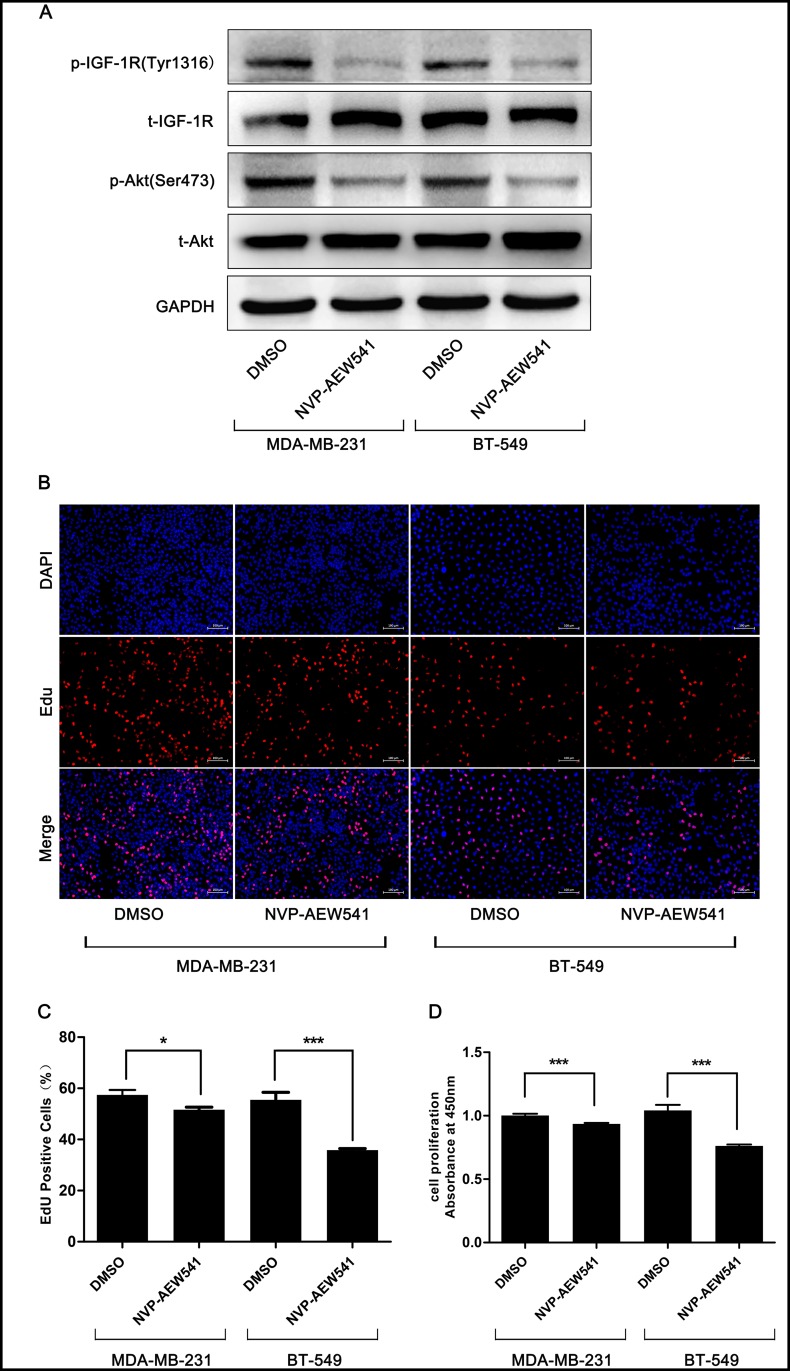
Effect of NVP-AEW541 on proliferation and levels of IGF-1R and Akt in MDA-MB-231 and BT-549 cells. (A) Western blotting validated that NVP-AEW541 significantly reduced expression of phosphorylated IGF-1R and Akt. One representative from three independent experiments is shown. (B-D) NVP-AEW541 markedly decreased the proliferation rate of MDA-MB-231 and BT-549 cells, as assessed by EdU (B and C) and cell counting kit-8 assays (D). Results were obtained from at least four independent experiments, and data are presented as the mean ± SD, *p<0.05, **p<0.01, ***p<0.001.

To investigate the effect of IGF-1R on TNBC cells, we chose a selective inhibitor of IGF-1R, NVP-AEW541[21], to down-regulate the level of phosphorylated IGF-1R in MDA-MB-231 and BT-549 cells. Western blotting confirmed the effect of NVP-AEW541 on phospho-IGF-1R (Fig 1A). EdU and CCK-8 assays were performed to examine the effect of NVP-AEW541 on cell proliferation, and the results showed that treatment with 1 μmol/L NVP-AEW541 for 24 h significantly reduced TNBC cell proliferation (Fig 1B–1D). The PI3K-Akt pathway is downstream of IGF signaling and related to autophagy [11]. Our results showed that the level of activated Akt, an autophagy inhibition gene [22], was also reduced by NVP-AEW541 (Fig 1A), suggesting that IGF-1R inhibition might induce autophagy in TNBC cells.

### NVP-AEW541 induced autophagy in MDA-MB-231 and BT-549 cells

**Fig 2:**
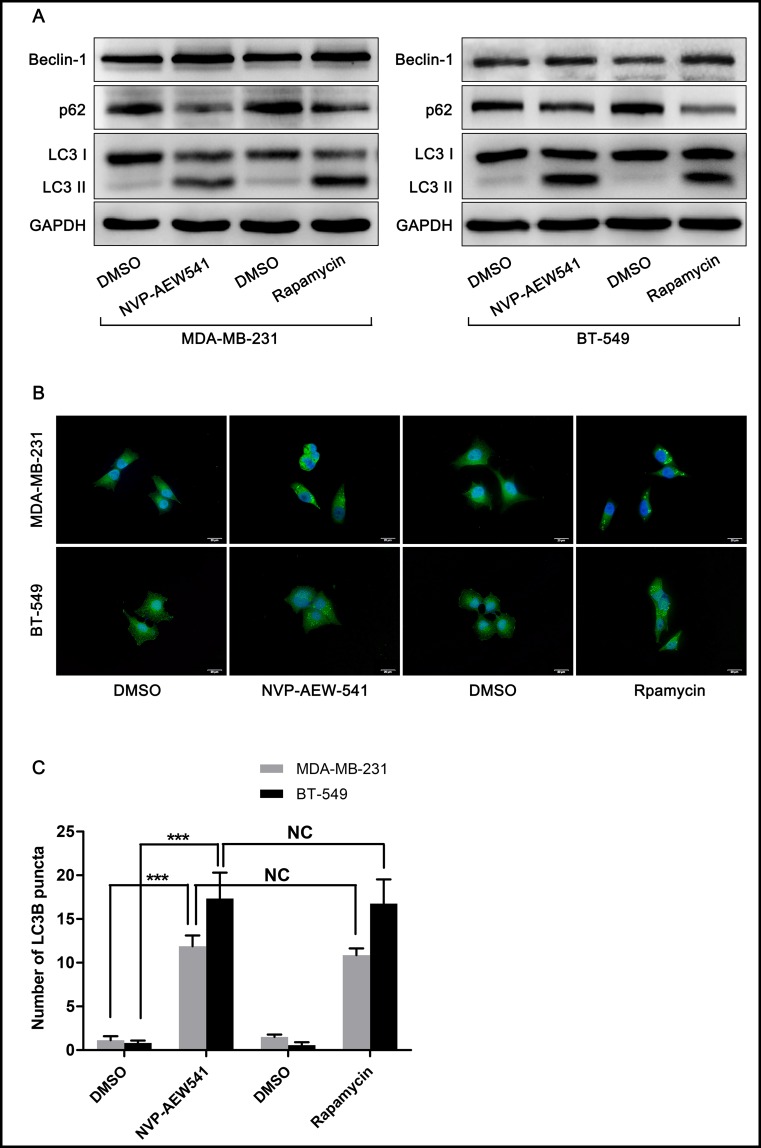
NVP-AEW541 induces autophagy in MDA-MB-231 and BT-549 cells. (A) NVP-AEW541 induced a significant accumulation of LC3-II and Beclin-1 and down-regulated p62 in MDA-MB-231 and BT-549 cells, as measured by western blotting. One representative from three independent experiments is shown. (B and C) Immunostaining and fluorescence microscopy analysis of LC3 (green) and the nucleus (blue) demonstrated that NVP-AEW541 promoted autophagy, as indicated by increased LC3-II puncta. Results were obtained from four independent experiments, and data are presented as the mean ± SD, *p<0.05, **p<0.01, ***p<0.001.

To confirm our hypothesis that IGF-1R inhibition might induce autophagy, rapamycin, an autophagy-promoting agent, was chosen as a positive control. As shown in Fig 2A, both NVP-AEW541 (1 μM) and rapamycin (10 nM) notably increased the expression level of the autophagy-related protein Beclin-1 and the ratio of LC3-II/LC-I and reduced the level of the selective autophagy substrate p62. The effect of NVP-AEW541 on LC3-II puncta formation was also tested. After treatment of cells with DMSO, NVP-AEW541 or rapamycin for 24 h, immunofluorescence analysis showed the remarkable accumulation of LC3-II puncta, a characteristic mammalian autophagy phenotype, in NVP-AEW541 or rapamycin treatment groups (Fig 2B and 2C). These results indicated that NVP-AEW541 could significantly induce autophagy in TNBC cells.

### Inhibition of autophagy enhanced NVP-AEW541-induced cell growth suppression in TNBC cells

**Fig 3:**
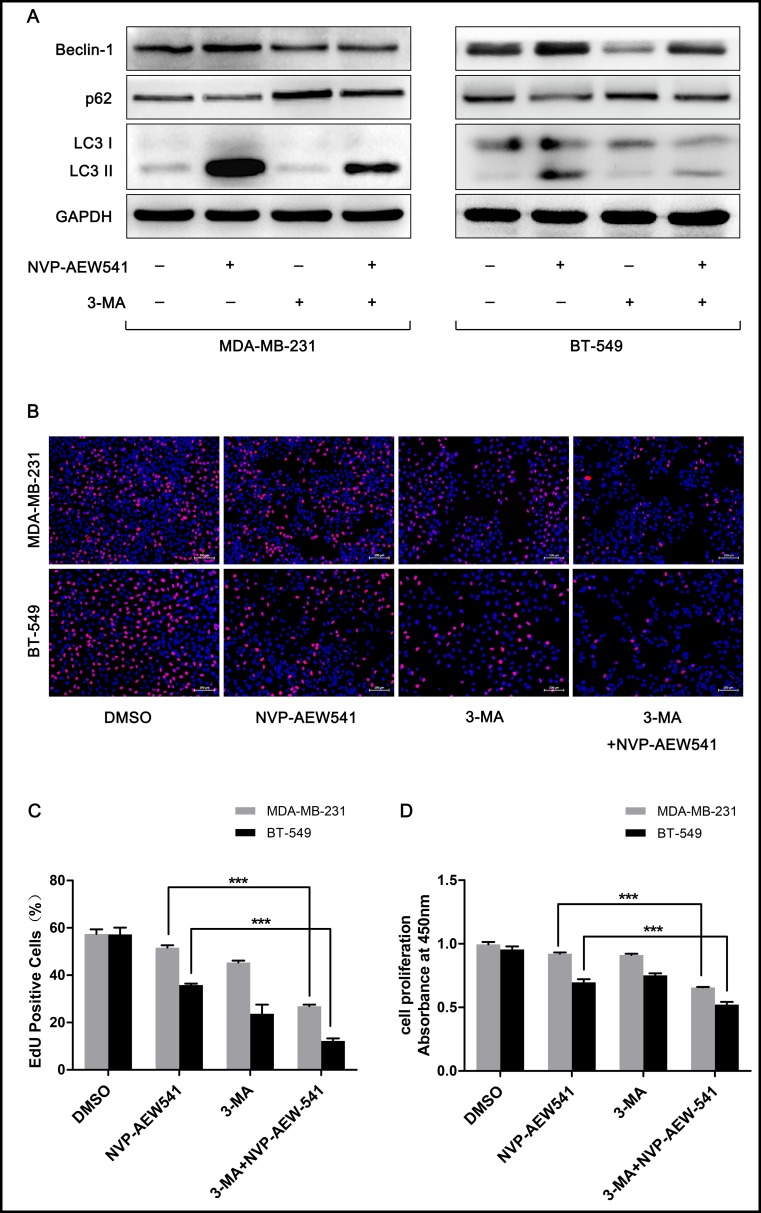
3-MA inhibits NVP-AEW541-induced autophagy and enhances anti-proliferative effects of NVP-AEW541 in MDA-MB-231 and BT-549 cells. (A) 3-MA inhibited the autophagy induced by NVP-AEW541 in MDA-MB-231 and BT-549 cells, as shown by western blotting. One representative from three independent experiments is shown. (B-D) 3-MA combined with NVP-AEW541 significantly suppressed the proliferation of MDA-MB-231 and BT-549 cells compared with NVP-AEW541 alone, as detected by EdU (B and C) and CCK-8 assays (D). Results were obtained from at least four independent experiments, and data are presented as the mean ± SD, *p<0.05, **p<0.01, ***p<0.001.

**Fig 4:**
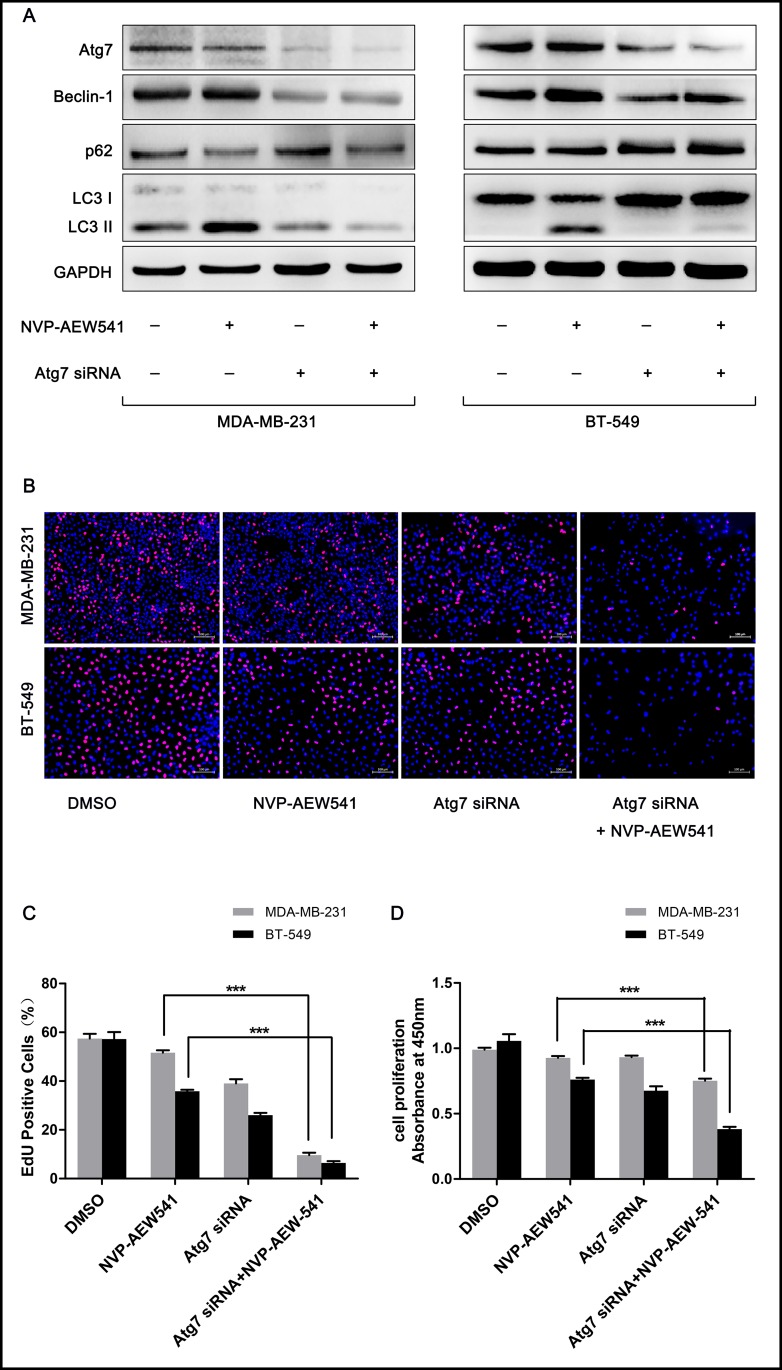
Atg7 siRNA enhances the inhibition of proliferation by blocking NVP-AEW541-induced autophagy in MDA-MB-231 and BT-549 cells. (A) Atg7 siRNA blocked NVP-AEW541-induced autophagy in MDA-MB-231 and BT-549 cells, as assessed by western blotting. One representative from three independent experiments is shown. (B-D) Atg7 siRNA combined with NVP-AEW541 significantly suppressed the proliferation of MDA-MB-231 and BT-549 cells compared with NVP-AEW541 alone, as detected by EdU (B and C) and CCK-8 assays (D). Results were obtained from at least four independent experiments, and data are presented as the mean ± SD, *p<0.05, **p<0.01, ***p<0.001.

To explore whether NVP-AEW541-induced autophagy is beneficial for TNBC cells, we combined the autophagy inhibitor 3-MA or siRNA targeting the autophagy-promoting gene Atg7 and examined the effects of NVP-AEW541 on TNBC cells. Western blotting analysis verified the autophagy-suppressive effect of 3-MA and Atg7 siRNA: Beclin-1 and the LC3-II/LC3-I ratio were reduced, and the level of p62 was enhanced (Fig 3A and Fig 4A). Compared with the NVP-AEW541 group, cells treated with NVP-AEW541 together with 3-MA or Atg7 siRNA showed lower levels of autophagy (higher Beclin-1 and LC3-II/LC3-I ratio and lower p62 level) (Fig 3A and Fig 4A). CCK-8 and EdU assays showed that NVP-AEW541 combined with 3-MA or Atg7 siRNA treatment significantly suppressed cell proliferation compared to NVP-AEW541 alone (Fig 3B–3D and Fig 4B–4D). These findings suggest that NVP-AEW541-induced autophagy might protect TNBC cells from growth suppression.

### Inhibition of autophagy enhanced NVP-AEW541-induced apoptosis in TNBC cells

**Fig 5:**
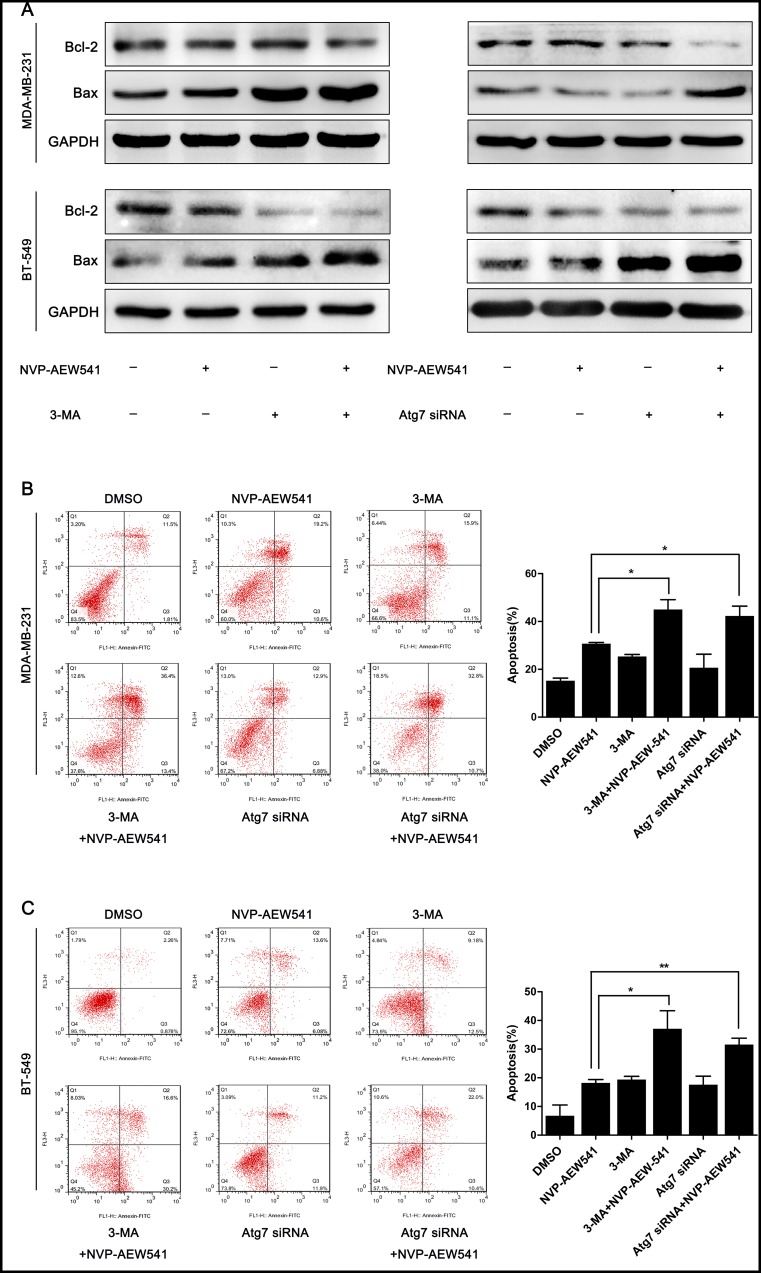
3-MA and Atg7 siRNA increase the level of apoptosis in MDA-MB-231 and BT-549 cells. (A) 3-MA and Atg7 siRNA decreased the protein level of Bcl-2 and increased that of Bax in MDA-MB-231 and BT-549 cells, as shown by western blotting. (B) 3-MA and Atg7 siRNA markedly increased the level of apoptosis induced by NVP-AEW541 in MDA-MB-231 cells, as determined by Annexin V/PI dual staining. (C) 3-MA and Atg7 siRNA markedly increased the level of apoptosis induced by NVP-AEW541 in BT-549 cells, as determined by Annexin V/PI dual staining. All experiments were repeated three times, and data are presented as the mean ± SD, *p<0.05, **p<0.01, ***p<0.001.

As the results presented above revealed that NVP-AEW541-induced autophagy protects TNBC cell proliferation, we then examined the effect of NVP-AEW541-induced autophagy on apoptosis. The expression levels of apoptosis-related proteins Bcl-2 and Bax were examined by western blotting analysis, and Annexin V/PI dual staining was performed to evaluate the rate of apoptosis. As shown in Fig 5A, cells exposed to NVP-AEW541 together with 3-MA or Atg7 siRNA presented higher Bax levels and lower Bcl-2 levels compared with the NVP-AEW541 group. Annexin V/PI dual staining revealed that NVP-AEW541 combined with 3-MA or Atg7 siRNA treatment increased the apoptosis rate from 30.8% to 45.0% and from 30.8% to 42.4%, respectively, in MDA-MB-231 cells and from 18.2% to 37.1% and from 18.2% to 31.6%, respectively, in BT-549 cells (Fig 5B and 5C). These results indicated that inhibition of autophagy enhances NVP-AEW541-induced apoptosis in TNBC cells.

### Inhibition of autophagy enhanced NVP-AEW541-induced cell cycle arrest in TNBC cells

**Fig 6:**
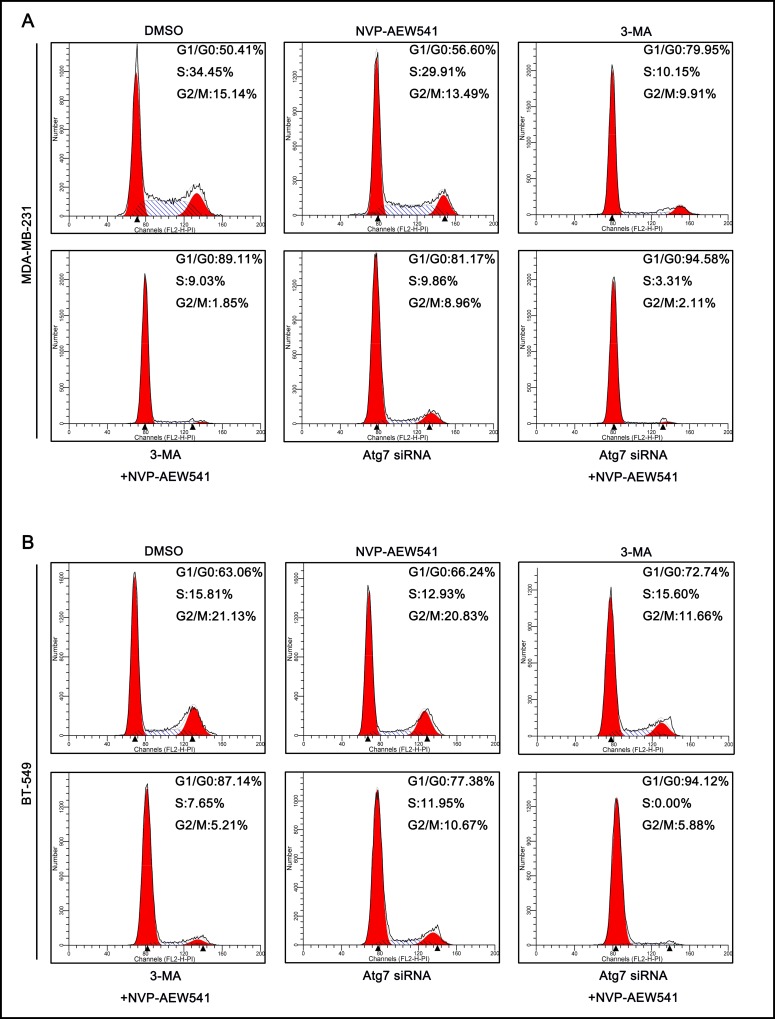
3-MA and Atg7 siRNA enhanced G0/G1 cell cycle arrest in MDA-MB-231 and BT-549 cells. (A) Either 3-MA or Atg7 siRNA combined with NVP-AEW541 induced a higher level of G0/G1 cell cycle arrest in MDA-MB-231 cells, as examined by flow cytometry. (B) Either 3-MA or Atg7 siRNA combined with NVP-AEW541 induced a higher level of G0/G1 cell cycle arrest in BT-549 cells, as examined by flow cytometry. One representative from two independent experiments is shown.

Because NVP-AEW541-induced autophagy enhanced the cell growth-suppressing effect of NVP-AEW541 in TNBC cells, we further investigated its effect on the cell cycle. Cell cycle analysis revealed that NVP-AEW541 slightly increased the proportion of cells in G1/G0 phase and lowered the number of cells in other phases compared with control group. In addition, compared with NVP-AEW541 alone, both 3-MA and Atg7 siRNA markedly enhanced the effect of NVP-AEW541 on MDA-MB-231 and BT-549 cell cycles, with higher G1/G0-phase arrest, as examined by flow cytometry (Fig 6A and 6B). These findings suggested that NVP-AEW541-induced autophagy might play a role in protecting TNBC cells from cell cycle arrest.

## Discussion

IGF-1R is a member of the insulin receptor (IR) family, which includes the IR, IGF-1R, IGF-1R/IR hybrid receptors and IGF-2R. Activating IGF-1R by IGF-1 or IGF-2 binding has an important function in tumorigenesis. Indeed, it has been reported that phosphorylated IGF-1R/1R is detected in all breast cancer subtypes (luminal, 48.1%; TNBC, 41.9%; HER2, 64.3%), with higher mortality [23]. A large portion of triple-negative breast cancer cells express IGF-1R, and activated IGF signaling promotes cell survival and proliferation [16]. These findings show that IGF signaling is a potential target for TNBC and other types of cancer. Therefore, efforts are being made to develop agents that target IGF-1R for cancer treatment, and several such drugs have been assessed in clinical trials [24]. There are two types of IGF-1R-targeted agents: monoclonal antibodies against IGF-1R, such as Dalotuzumab [25] and Ganitumab (AMG 479) [26]; and IGF-1R kinase inhibitors, including Linsitinib, and BMS-754807 [27]. Despite early accomplishments in pre-clinical investigations, in clinical trials, relatively few tumor types, e.g., Ewing sarcoma [28] and thymoma [29], exhibited sustained responses to IGF-1R inhibitors. Thus, IGF-1R inhibitors have yet to show satisfactory clinical benefit to patients with more prevalently diagnosed cancers, including breast cancer and non-small cell lung cancer, in the overall patient population [30, 31].

The complexity of IGF signaling may be one of the reasons for the failure of IGF-1R-targeted agents in clinical trials involving many types of cancers. Moreover, similar to IGF-1R interaction with IGF-1, binding of IGF-2 to IGF-1R or IR-A can also stimulate IGF signaling. The situation is further complicated when cells contain hybrid heterodimeric receptors consisting of IGF-1R and insulin receptor subunits, which can act as a major transducer of IGF signaling [32]. In addition, HIF-1-induced IGF-2 release and compensatory activation of IR was also reported in TNBC cells under hypoxic conditions when IGF-1R was specifically targeted, thus displaying the function of IGF-1 signaling [15].

Autophagy is a lysosomal degradation process that can be stimulated by various stresses such as starvation, hypoxia or infection. Autophagy is involved in many diseases, including cancers. It has been reported that IGF-1 regulates autophagy through c-jun N-terminal kinase and Akt pathways in human atherosclerotic vascular smooth cells [33]. However, it has not yet been elucidated whether IGF-1R inhibition can induce autophagy in TNBC cells. In this study, we found that IGF-1R inhibition by NVP-AEW541 could not only inhibit cell proliferation but also suppress the PI3K-Akt pathway in TNBC cell lines MDA-MB-231 and BT-549 (Fig 1). In addition to regulating cell survival, proliferation and metabolism, the PI3K-Akt pathway, a well-known pathway downstream of IGF signaling, also participates in autophagy by regulating the autophagy modulator mTORC-1 [11]. Accordingly, we further examined whether IGF-1R inhibition would alter the autophagy level. After treatment with NVP-AEW541, both MDA-MB-231 and BT-549 cells showed increased levels of autophagy-related protein Beclin-1 as well as the LC3-II/LC3-I ratio and decreased levels of the selective autophagy substrate p62 (Fig 2A). Furthermore, LC3-II puncta, a well-characterized phenotype indicating autophagy, were remarkably accumulated after NVP-AEW541 treatment (Fig 2B and 2C). These results indicate that IGF-1R inhibition by NVP-AEW541 can suppress cell proliferation and significantly induce autophagy in TNBC cells.

The consequence of autophagy on cancer is ambiguous and depends on the cell context and stage of tumor progression [11]. Decreases in autophagy have been found in the most aggressive malignant hepatocellular carcinoma (HCC) cell lines and HCC tissues with recurrent disease, revealing a tumor-suppressive function for autophagy in HCC [34]. In contrast, reductions in autophagy delay tumor formation and reduce tumorigenesis in breast cancer models [35, 36]. In view of the double-sided effects of autophagy in cancer, we set forth to investigate whether IGF-1R inhibition-induced autophagy is beneficial to TNBC cells. We found that autophagy inhibition enhanced the suppression of cell growth and apoptosis induced by NVP-AEW541. These results suggested a protective role for IGF-1R inhibition-induced autophagy in TNBC cells. This may, to some extent, account for the lack of clinical efficacy of IGF-1-targeting agents in breast cancer and even in other cancers such as lung cancer. Therefore, our study may provide a valuable treatment strategy for IGF-1R inhibitor-based therapies by combining the use of autophagy-disrupting agents for the treatment of IGF-1 signaling-associated tumors.

To explore the mechanism by which NVP-AEW541-induced autophagy protects TNBC cells, we sought to examine changes in apoptosis-related proteins Bax and Bcl-2 after inhibiting IGF-1R. Western blotting analysis showed that combining NVP-AEW541 with 3-MA or Atg7 siRNA markedly up-regulated the level of Bax expression while simultaneously down-regulating that of Bcl-2 expression compared with NVP-AEW541 alone. Bax and Bcl-2 belong to two groups of BCL-2 family proteins, known as proapoptotic and antiapoptotic proteins, respectively, which mediate mitochondrial apoptosis by modulating mitochondrial outer membrane permeabilization [37]. This result indicated that IGF-1R inhibition-induced autophagy might modulate apoptosis of TNBC cells by regulating the levels of Bax and Bcl-2 expression.

In conclusion, our study indicates that IGF-1R inhibition can induce TNBC cell-protective autophagy, which may partially recuse cells from the proliferation suppression and apoptosis caused by IGF-1R inhibition and thereby weaken the efficacy of IGF-1R-targeting agents. Autophagy-disrupting agents can enhance the effect of IGF-1R inhibitors and will be a valuable complement to IGF-1R inhibitor-based therapies for cancers. This may constitute a potential therapeutic strategy for TNBC.
